# Dufourmentel Flap for Scalp Reconstruction

**DOI:** 10.1097/GOX.0000000000004183

**Published:** 2022-09-20

**Authors:** Enrique J. Viciana, Anne-Sophie Lessard

**Affiliations:** * Formerly of Mercy Hospital, Department of Surgery, Division of Plastic Surgery; Private Practice, Miami, Fla.; † Plastic Surgery Boutique, Miami, Fla.

## Abstract

Transposition flaps are useful for reconstruction of many skin defects. Limberg described a rhomboid rotation flap in 1946. Dufourmentel described an improved version of the Limberg flap in 1962. The Dufourmentel flap is also a quadrangular rhomboid flap which can be used in any area of the body except for the central face, the fingers, and the volar hand. The design of the Dufourmentel flap creates a wider base for this random flap, thus making it more reliable. Where tissue loss is significant, or where skin and soft tissue elasticity is limited, double opposing Dufourmentel flaps are useful. A variation of the Dufourmentel flap is described where a circular defect is converted to a square. The line of greatest extensibility is marked through the circular defect or lesion. A square is marked around the circle with one corner of the square tilted 10–20 degrees counterclockwise from the line of greatest extensibility. After marking corners A, B, C, and D, lines are marked extending BD and CD. The first incision, DE, will bisect the angle created by extending BD and CD. The second incision, EF, is roughly perpendicular to CD extended, but the angle at E is opened up a bit to create a wider base for the flap. Point D will rotate to point B, E rotates to C, and F translates to D. The invisible line DF should be approximately parallel to the line of greatest extensibility. When the defect is relatively large or where the surrounding tissues have limited elasticity, the above-described ideal variation of the Dufourmentel flap may not be possible because the flap may not rotate and advance all the way around without tension. In this case, double opposing Dufourmentel type flaps have been found to be useful by meeting each other at the halfway point.

**Takeaways**

**Question:** How to reconstruct a large scalp defect with exposed cranium?

**Findings:** We describe the surgical anatomy, the surgical technique, and a case example of the double opposing galeocutaneous modified Dufourmentel flaps for reconstruction of a large scalp defect with exposed cranium.

**Meaning:** The double opposing Dufourmentel flap is a reconstructive method that is useful when a large defect is difficult to close with a single transposition flap or when donor tissue is limited or has limited elasticity.

## INTRODUCTION

Rhomboid cutaneous flaps have been used successfully for reconstruction of the breast and chest wall.^1^ Rhomboid opposing cutaneous flaps have been used for reconstruction of the nasal dorsum, albeit with extensive scarring,^2^ lateral forehead,^3^ and oral commissure.^4^ Double opposing cutaneous flaps have been used successfully for shoulder and for medial thigh reconstruction,^5^ and double opposing galeocutaneous flaps have been described for reconstruction of a large aplasia cutis congenita defect in a neonate.^6^ Double opposing rhomboid fasciocutaneous flaps have been used successfully for large defects following excision of extensive complex pilonidal sinus disease.^7^ We describe double opposing galeocutaneous modified Dufourmentel flaps for reconstruction of a large scalp defect with exposed cranium.

Claude Dufourmentel (1915–2012) was a French plastic surgeon who described a modification of the Limberg flap in 1962.^8^ The Limberg rhomboid flap was previously described by Alexander Limberg (1894–1974), a Russian surgeon, in 1946.^9^ Dufourmentel’s improved design gives this random flap a wider pedicle, thus making it more predictable. The geometric principles of the Dufourmentel flap are as follows:

1. Convert the defect into a rhombus with angles of 60 degrees and 120 degrees.
2. Make the first incision bisecting half of the obtuse angle, AE
3. After making a line through the obtuse angle, make the second incision perpendicular to this line‚ EF.

As our defect was large and the donor tissue available had limited elasticity, the flap design was based on conversion of the defect to a square. The flaps were drawn so the line of translation of each flap would be parallel to the line of greatest extensibility. Two double opposing flaps were designed. Dissection was made deep to the galea. Scoring of the galea was not performed.

## INDICATIONS AND CONTRAINDICATIONS

The double opposing Dufourmentel flap is a reconstructive method that is useful when a large defect is difficult to close with a single transposition flap or when donor tissue is limited or has limited elasticity. The method can be applied anywhere except the central face, such as the columella,^10^ the fingers, and the volar hand. These flaps may be cutaneous or fasciocutaneous. When cutaneous, they are generally random flaps. When fasciocutaneous, they may be axial or random.

Facial defects are poorly closed with this technique or with any transposition flap, due to the lack of attention to aesthetic subunits, to the ensuing secondary deformity, and to the extensive scarring. Large defects of the cheek or chin are often best reconstructed with skin grafts when a reverse facelift procedure cannot close the defect. Finger and hand defects may be closed with skin grafts, cross finger flaps, V-Y flaps, or thenar flaps.

In poor surgical candidates, skin grafting or normal saline dressing changes with healing by secondary intent should strongly be considered. Medial canthal defects often heal surprisingly well when allowed to heal by secondary intent.^11^ Conditions that may lead to poor healing include Raynaud’s disease, severe peripheral vascular disease, scleroderma, systemic lupus erythematosus, dermatomyositis, and also with calcinosis, Raynaud’s phenomenon, esophageal dysfunction, sclerodactyly, and telangiectasia syndrome. In patients with diabetes mellitus with vascular involvement, consideration should be given to a surgical delay of the flap by incising through skin only and completion of the flap at 14 days.^12^

When closing areas with exposed vital tissues or exposed bone, or areas at risk for contamination such as pilonidal disease, fasciocutaneous flaps are more robust and, therefore, more protective than cutaneous flaps.

## SURGICAL ANATOMY

The galea aponeurotica is a layer of dense tendon-like tissue that connects the frontalis and occipitalis muscles. It is innervated and vascularized from branches arising in the frontalis and occipitalis muscles. The skin, subcutaneous tissue, and galea are often considered a single layer. Dissection in the loose areolar tissue between the galea and pericranium glides easily.

## SURGICAL TECHNIQUE

1. The procedure is performed under general anesthesia or with diluted local anesthesia and intravenous sedation.
2. Generous padding is placed under all pressure points.
3. The sequential compression device is applied and tested. If the legs are not available for the sequential compression device, it may be placed on an upper extremity.
4. The head is elevated 30 degrees.
5. Infiltrate the incisions and soft tissues with diluted 1% lidocaine with epinephrine 1:100,000. Wait 10 minutes.
6. The wound and surgical sites are irrigated with normal saline.
7. Nonviable areas of skin, fat, fascia, and bone are debrided and sent to pathology.
8. The line of greatest extensibility is drawn through the center of the circular defect with a marking pen.
9. A square is marked around the circle with one corner tilted 10–20 degrees counterclockwise from the line of greatest extensibility.
10. Mark the Dufourmentel type flaps with surgical ink in a double opposing fashion.
11. Imaginary lines DF and BF’ should be approximately parallel to the line of greatest extensibility.
12. Make the incisions and rotate and advance the flaps into position without tension.
13. The galea may need to be underscored without disturbing its blood supply to obtain tension free rotation advancement.
14. When incising through the scalp, Raney clips should be applied to obtain temporary hemostasis. The clips are removed sequentially as the galea and skin are closed.
15. Temporary hemostasis is also obtained with pressure applied by the assistant surgeon. Visible divided vessels should be controlled by grasping with the fine Adson forceps with teeth and touching the cautery to the forceps. At no time should cautery be touched directly to the tissues.
16. Secure the galeal aponeurosis with inverted interrupted sutures of 4-0 Vicryl.
17. Suture the skin with interrupted sutures of 4-0 Prolene and 5-0 Nylon. With dark skin use 4-0 Prolene. Prolene is useful in areas of mild tension because it can stretch.
18. It is acceptable to leave gaps between the sutures for drainage and to prevent tension.
19. Cleanse the wounds and surgical sites with normal saline.
20. Apply a dressing of Xeroform gauze and gauze 4 × 4’s and secure it in place with a modified Russian-style bandage^13^ using a Kerlix gauze and 4 × 4’s.

## POSTOPERATIVE CARE

The patient should be seen in the office at 48 hours or at 24 hours if drains are present. A new sterile dressing should be applied at the first postoperative visit, as the incisions are still open wounds and should be protected. If a Penrose drain is used, it should usually be removed at 48 hours. With a Jackson-Pratt drain, removal is when a certain threshold is crossed, such as less than 10 cm^3^ of drainage in 24 hours. The patient is seen again on postoperative day 5. The dressings are removed, and the wounds and sutures are left open to air. Sterile dressings should be applied to the site of a Jackson-Pratt drain until the opening is sealed. The patient is instructed to begin washing the surgical area daily with soap and water. If the double opposing fasciocutaneous Dufourmentel flaps are not on the face, the sutures should remain for 10–14 days. Facial sutures should be removed after 5 days. Activities should be limited for the first postoperative month. Sunblock should be used for 6–12 months.

## PEARLS AND PITFALLS

Standing cones should never be excised preemptively, as the size and shape of the standing cone is unpredictable. Additionally, this excess tissue may be needed. All plastic surgeons are familiar with excision of a standing cone.

Antibiotics active against methicillin-resistant *staphylococcus aureus* should be prescribed to begin the evening before surgery and continued for only 24 hours. In this case, the wound contained necrosis, and the patient was elderly; therefore, the antibiotics were continued for 5 days. The surgeon should prescribe Duricef 500 mg daily or for those with penicillin allergy, Levaquin 500 mg daily. Clearly written instructions should be given to the patient, preoperatively if possible.

With the excision of the square surrounding the circular defect, the excision at points A and C should be curved to facilitate closure. The patient should be advised that all incisions leave scars and that these scars are permanent.

Bleeding scalp edges should not be cauterized, as this will create fat necrosis without achieving hemostasis.

The use of allografts or bioengineered materials should be avoided because they are frequently rejected as foreign bodies.

## COMPLICATIONS

All flaps may fail. The two most common reasons for flap failure are torsion of the pedicle and tension when insetting the flap. Torsion of the pedicle can result in loss of the entire flap. Flap loss due to tension is more common, and the amount of tissue loss is variable. What is nearly always true is that tension will cause necrosis of the distal flap, which is usually the most necessary portion of the flap. Most Dufourmentel fasciocutaneous flaps are random flaps, but some may be axial flaps. If the pedicle of the flap is parallel to the fibers of the underlying muscle, then the flap is likely to be an axial flap and will have a more robust blood supply than a random flap. These axial flaps are much less likely to fail. Edge necrosis may be seen with the use of nicotine products.

Infections are uncommon with fasciocutaneous flaps due to their vigorous blood supply. When infections do occur, they can be usually traced to inadequate debridement of the wound or to misuse or overuse of cautery. When cautery creates significant areas of necrosis, the necrosis becomes a nidus for infection as circulating bacteria attach themselves to the necrotic area. Use of cautery should be kept to a minimum, the temperature of the cautery should not be too high, and the cautery tip should always touch a forceps, but never the soft tissue directly. Treatment of a flap cellulitis requires intravenous antibiotics under the direction of an infectious disease specialist. If improvement is not rapid, treatment may also require debridement of all necrotic tissue, a process that may require re-elevation of the flaps for identification of necrotic tissue, followed by debridement and thorough irrigation. If purulence is present, a culture should be taken immediately. A return to the operating room for a formal incision and drainage should strongly be considered. Debridement of necrotic tissue may be necessary, which may also necessitate re-elevation of the flaps.

Hematomas may occur due to inadequate hemostasis or due to inadequate drainage of a surgical site. A surgical bed does not need to be completely dry for hemostasis to be adequate. A small amount of oozing is acceptable and will be reabsorbed by the soft tissues. But larger surgical sites have larger vessels and thus pose a greater risk of hematoma. In general, the larger the surgical site, the greater will be the need for drainage. Penrose drains are appropriate for only small wounds. Closed suction drains such as Jackson-Pratt drains are superior in efficacy and can also quantify the amount of drainage.

## INTRODUCTION/CASE EXAMPLE

A 78-year-old otherwise healthy White man presented to his dermatologist with a neglected ulcerating tumor of the vertex of his scalp. A biopsy demonstrated well-differentiated squamous cell carcinoma.

He was referred to the Mohs surgeon for micrographic excision of the carcinoma. After obtaining clear margins, the patient was referred to us for plastic surgical reconstruction.

The patient’s appearance was consistent with his stated age. His affect and cognition were normal. The review of systems was noncontributory. HEENT examination was within normal limits. Examination of the head and neck revealed no lymphadenopathy. The scalp appeared healthy with thinning light brown and white hair and no additional lesions.

The wound was surrounded by healthy skin with good sensibility and no cellulitis. There was no purulence in the wound or discoloration. The diameter of the defect was approximately 2.5 cm, with a depth of 1.5 cm.

**Fig. 1.**
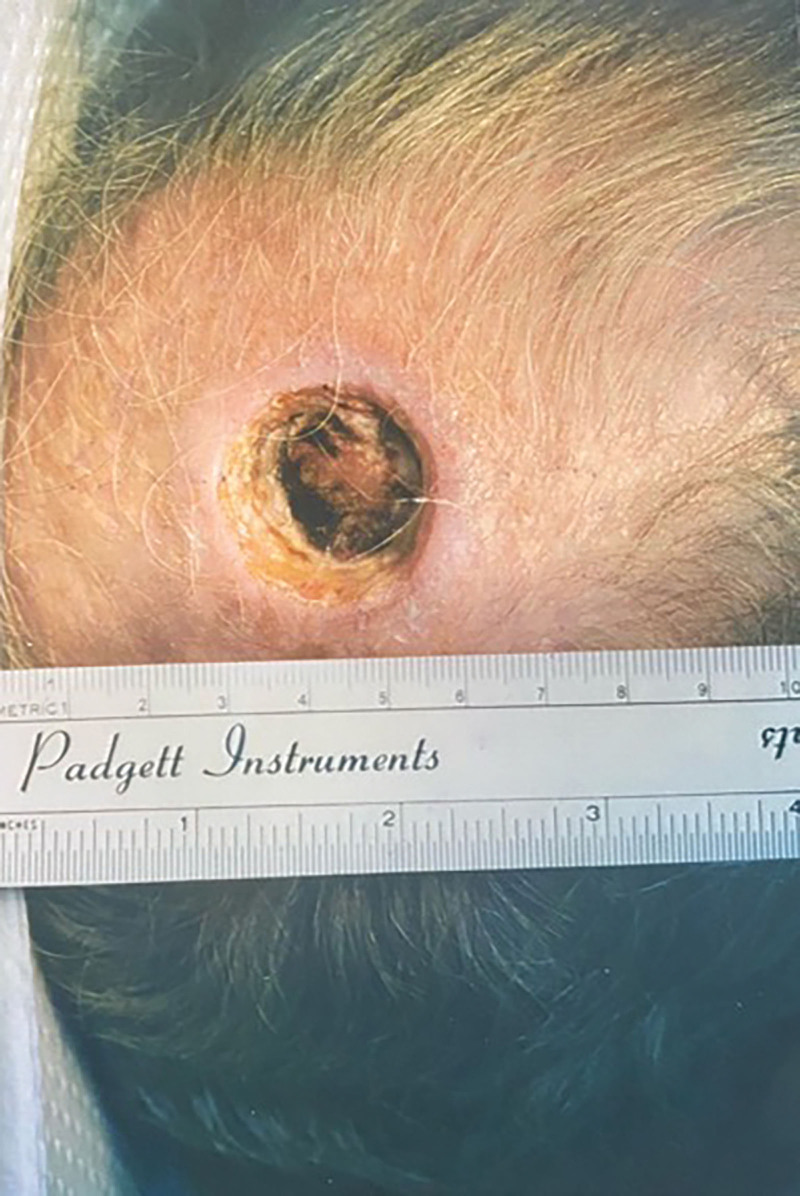
A 78-year-old man presented with this defect of the scalp vertex following Mohs excision of a squamous cell carcinoma.

The sides of the wound demonstrated cauterized, necrotic fat and fascia. The base of the wound was composed of necrotic pericranium over the calvaria. There was no bleeding (Fig. 1). A normal saline wet-to-dry dressing was applied.

Options for treatment of the wound were discussed in detail with the patient and with his family. They understood, asked questions, and all of their questions were answered. Conservative management consisting of daily normal saline wet-to-dry dressing changes followed by skin grafting if necessary was discussed. The area of exposed cranium could possibly need burring to accept a skin graft. Plastic surgical flap reconstruction could be accomplished with a large rotation flap with skin grafting of the donor defect or with bilateral opposing flaps such as V-Y flaps or Dufourmentel flaps with simultaneous closure of incisions. Diagrams of the flap approaches were shown to the patient and his family and they opted for the last-mentioned plan.

**Fig. 2.**
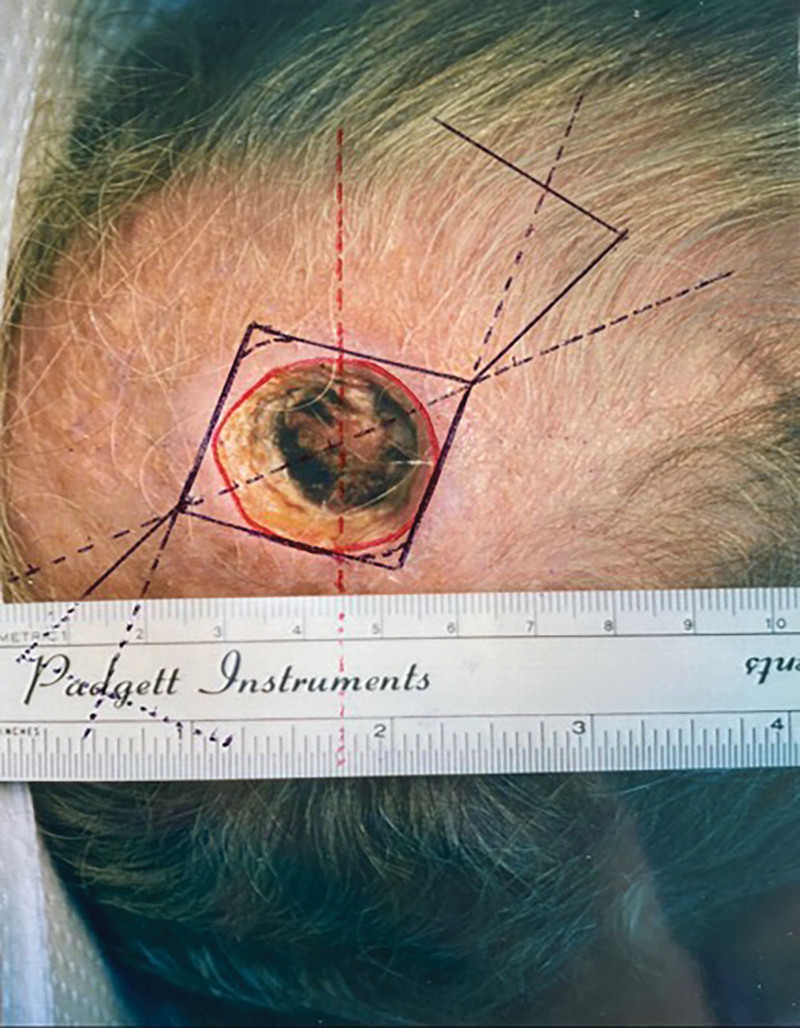
Double opposing Dufourmentel-style flaps were designed for closure of the defect.

**Fig. 3.**
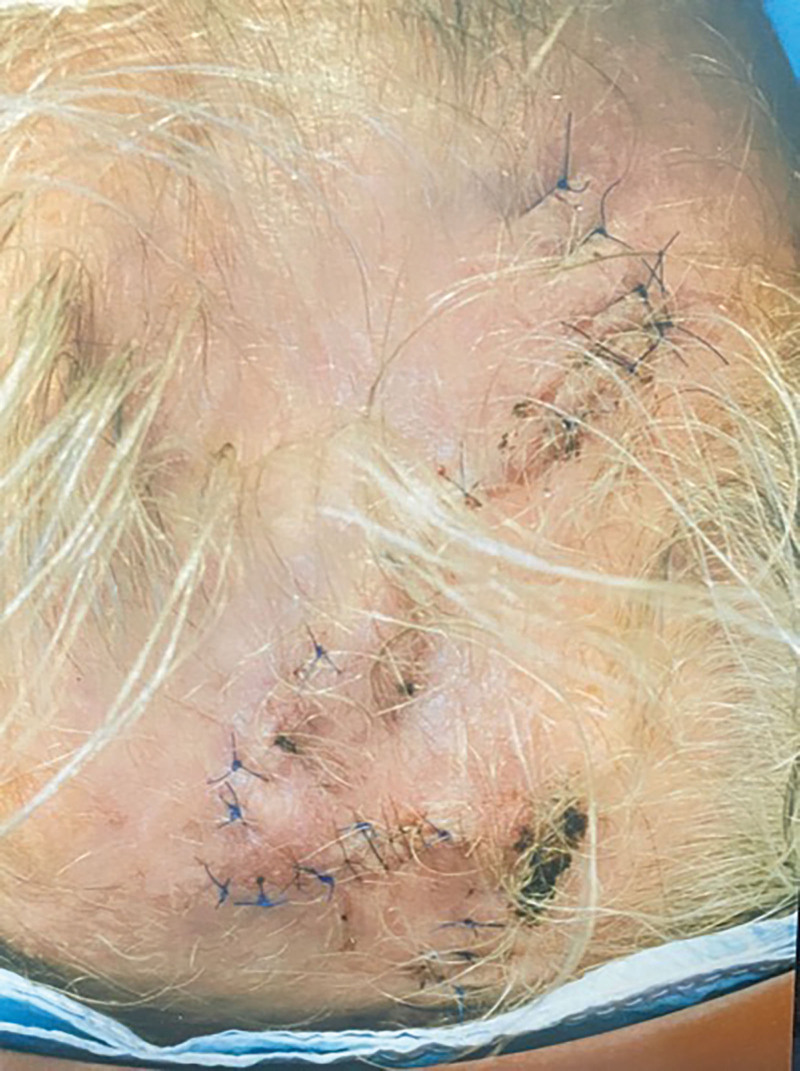
This is the appearance of the area on postoperative day 12.

With proper consent, photographs of the patient were taken by the surgeon of record preoperatively and 12 days postoperatively. A medical clearance was obtained before surgery. The circular defect was converted to a square and double opposing modified Dufourmentel type flaps were designed (Fig. 2). All excised tissue was sent to pathology, with a suture marking the 12 o’clock position. Excellent results were obtained without standing cones. A small area of superficial eschar was seen on postoperative day 12 (Fig. 3). There was no infection or other complication. The superficial eschar was treated with daily normal saline dressing changes, and the area healed well.

A larger rotation flap was considered, but would have needed to be quite large to obtain the necessary rotation as a galeocutaneous flap without scoring the galea. In addition, a large rotation flap would have created a large donor defect and a large standing cone (“dog ear”). The donor defect could be skin grafted with skin from the standing cone but this would not be ideal. The design of the Dufourmentel flap automatically closes the donor defect and eliminates standing cones.

It is not unusual for the distal aspect of a flap to slough, creating the need for readvancement. In this case, readvancement of a rotation flap may be more straightforward but Dufourmentel flaps may also be readvanced. In both designs, the area to be closed is identical and so the amount of scarring is comparable.

## DISCUSSION

Reconstruction of the scalp poses difficult challenges for the plastic surgeon. The design of the reconstruction is affected by the dome-shaped anatomy of the calvaria, the presence of hairlines, the direction of hair growth, the thickness yet elasticity of the scalp, and the limitations on this elasticity by the galea, a fibrous aponeurosis.

In general, small defects up to 2 cm^2^ may be closed by primary closure or with bilateral tissue advancement. Moderate defects up to 25 cm^2^ may be closed with V-Y flaps or rotation-advancement flaps, either single or bilateral. Larger defects require Juri flaps or other bipedicle flaps, Orticochea flaps, or the use of tissue expanders. In extreme cases, a free tissue transfer may be necessary. The scalp vertex has a whorl pattern, which should be maintained if possible. An important advantage of scalp reconstruction is the absence of motion, obviating the need for splinting. Pinwheel flaps consisting of three adjacent rotation-advancement flaps can be used but create three donor defects and three standing cones.

Following debridement of the wound, the diameter of this defect increased to approximately 3 cm. A variety of reconstructive options were available for this defect of 7 cm^2^. Although located at the vertex of the scalp, the amount of thinning hair demonstrated no whorl pattern. Conservative management with daily normal saline dressing changes would be expected to produce not only granulation tissue but also some wound contracture. This contracture could potentially cover or nearly cover the calvaria. If the wound failed to reduce to cover the calvaria, burring of the outer table to obtain granulation tissue would be necessary. All open tissue would need skin grafting. This process could be expected to take several weeks or longer. The ideal skin graft would be a full thickness skin graft, most likely harvested from the inguinal crease.

Flap reconstruction is usually performed using galeocutaneous flaps, although flaps composed of pericranium with its overlying areolar tissue have been mentioned. V-Y flaps have also been mentioned, but the mobility of such flaps would seem to be limited.

Orticochea flaps could be considered, but would involve an overly extensive operation for a defect of this size. A large rotation flap would produce a desirable result, albeit with the need to skin graft the resultant donor defect (“backgrafting”). The resultant standing cone could be the donor site for the full thickness skin graft.

Ultimately, the method of double opposing modified Dufourmentel flaps was elected, with the consent of the patient and his family. Although this design has not previously been described for the scalp, it has been used successfully in other anatomical sites, as described above. This method affords many favorable advantages. The flaps are composed of reliable galeocutaneous tissues. No open donor sites or standing cones are created. Although a large rotation flap is possibly easier to readvance, the modified Dufourmentel flaps can also be readvanced.

Further details regarding scalp reconstruction are beyond the scope of this discussion, but can be found in encyclopedic papers in the plastic surgical literature.^14,15^

When closing a skin and soft tissue defect, primary closure of the defect is the first option. When the defect will not close, or when primary closure will create too much tension, a local transposition flap is the next option. Limberg and Dufourmentel flaps are transposition flaps that are geometrically designed to allow for primary closure of the donor defect.

**Fig. 4.**
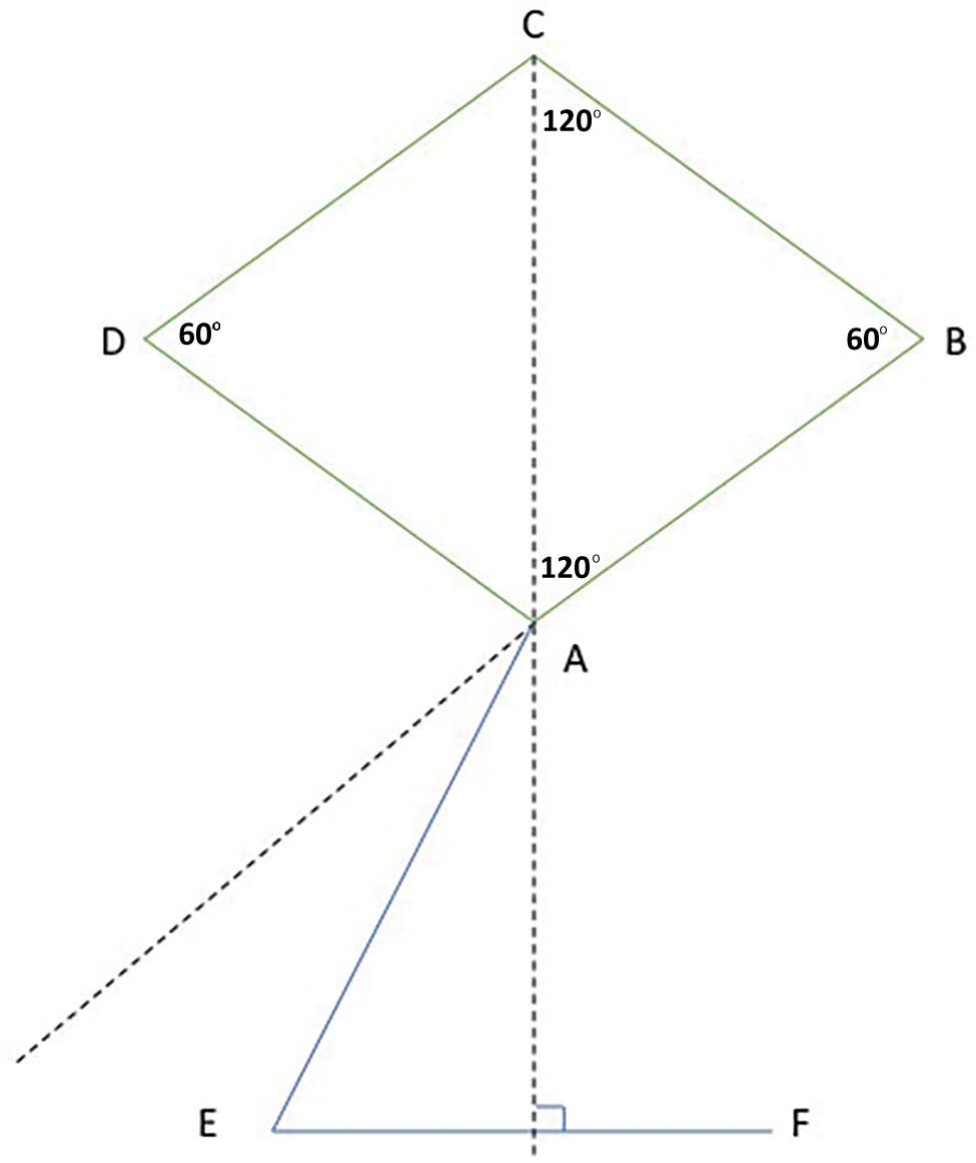
Depiction of the original design of the Dufourmentel flap.

In the Dufourmentel modification of the Limberg flap, a rhombus of equilateral sides is drawn around the lesion or defect with opposing angles of 60 degrees and 120 degrees. This rhombus is shown in Figure 4 with points ABCD. The first incision, AE, bisects the angle formed by AB extended and AC extended and is of equal length as the sides of the rhombus. The second incision, EF, is perpendicular to AC extended and is also of equal length. Point A will rotate to point C, E will rotate to D, and F translates to A. The invisible line AF should therefore be as parallel as possible to the line of greatest extensibility.

**Fig. 5.**
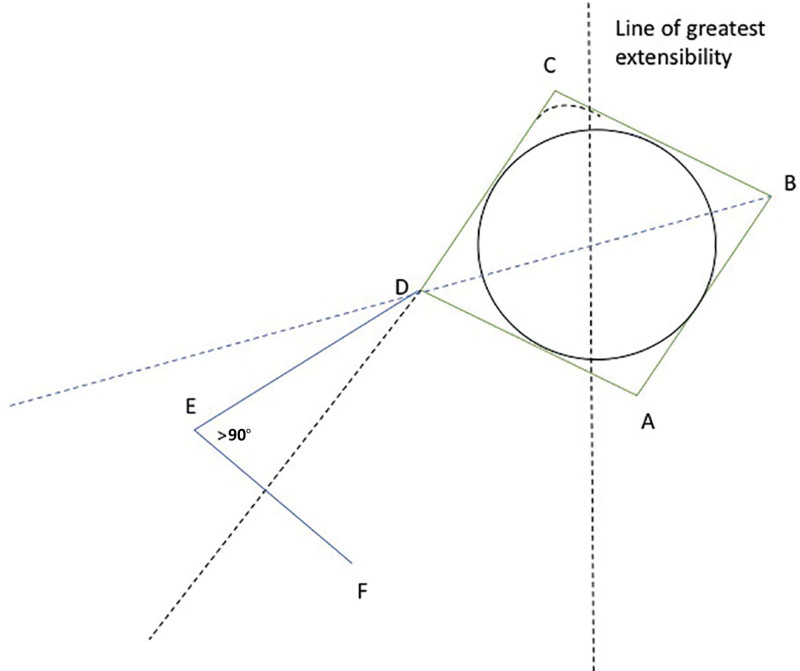
Depiction of the author’s modification of the Dufourmentel flap, where a circular defect is converted to a square.

When a circular defect is converted to a square, such as in this modification of a Dufourmentel flap, the line of greatest extensibility is drawn through the center of the circle (Fig. 5). The square is drawn with one corner tilted 10–20 degrees counterclockwise from the line of greatest extensibility. The angle at E is opened slightly to create a wider pedicle base. Curving the excision at C is helpful for closure.

When the defect is relatively large, or when the surrounding tissue has limited elasticity, double opposing modified Dufourmentel flaps are useful for closing the defect as seen in Figure 2. The flaps are designed as mirror image double opposing flaps. The excisions are curved at A and at C to facilitate closure.

It is important to design the double opposing Dufourmentel flaps so that imaginary lines DF and BF’ are as parallel as possible to the line of greatest extensibility. For this reason, there are only two ideal Dufourmentel flaps surrounding a circular defect. There cannot be an unlimited number of flaps available.^16^

## CONCLUSIONS

In this patient, an excellent result was obtained with bilateral opposing modified Dufourmentel flaps. Healthy fasciocutaneous units covered the defect in one stage without open donor sites or standing cones. The wounds demonstrated primary healing with only a small superficial eschar, which healed rapidly without infection. The location of the eyebrows was not distorted. The hairlines were maintained. Unfortunately, the patient was lost to follow-up. It is hoped that he continued to do well.
